# Light-Dependent and Circadian Transcription Dynamics *In Vivo* Recorded with a Destabilized Luciferase Reporter in *Neurospora*

**DOI:** 10.1371/journal.pone.0083660

**Published:** 2013-12-31

**Authors:** François Cesbron, Michael Brunner, Axel C. R. Diernfellner

**Affiliations:** University of Heidelberg Biochemistry Center (BZH), Im Neuenheimer Feld, Heidelberg, Germany; Karlsruhe Institute of Technology, Germany

## Abstract

We show that firefly luciferase is a stable protein when expressed at 25°C in *Neurospora*, which limits its use as transcription reporter. We created a short-lived luciferase by fusing a PEST signal to its C-terminus (LUC-PEST) and applied the LUC-PEST reporter system to record *in vivo* transcription dynamics associated with the *Neurospora* circadian clock and its blue-light photosensory system over the course of several days. We show that the tool is suitable to faithfully monitor rapid, but also subtle changes in transcription in a medium to high throughput format.

## Introduction

Firefly luciferase from *Photinus pyralis* is a highly sensitive reporter for monitoring gene expression activity in a variety of organisms [1], [2], [3], [4], [5]. In contrast to fluorescence, luciferase does not require light irradiation for excitation and is thus neither subject to photobleaching nor phototoxicity. Hence, luciferase is a suitable reporter for long term *in vivo* measurements that are extensively used to record and monitor circadian clock activity in bacteria, fungi, plants, flies and mammals [3], [6], [7], [8], [9].

Circadian clocks are self-sustained molecular oscillators that are dependent on interconnected transcriptional and posttranscriptional negative feedback loops [10]. In *Neurospora*, the circadian transcription factor WHITE COLLAR COMPLEX (WCC) controls rhythmic expression of FREQUENCY (FRQ), which in turn inhibits the WCC in a negative feedback loop [11], [12], [13], [14]. The WCC is also directly activated by light via its LOV-photoreceptor domain [15]. In light-dark cycles the activity of the WCC is additionally attenuated by the LOV-photoreceptor VIVID [16], [17], [18], which directly interferes with light-dependent dimerization of the WCC via LOV-LOV interactions [15]. These feedback loops support rhythmic transcription of *frq* and other clock-controlled genes in constant darkness and also in light-dark cycles.

A *Neurospora* reporter assay based on a codon-optimized luciferase gene allows to record circadian genes expression rhythms *in vivo* over the course of several days [19]. The stability of firefly luciferase is temperature dependent [20], [21]. In mammalian cells at 37°C luciferase is degraded with a half-time of about 3–4 h [22]. Addition of a PEST sequence significantly destabilizes luciferase [23], rendering it more suitable as a reporter for dynamic processes on a short time-scale.

We show here that luciferase is extremely stable (t_1/2_ ∼ 8 h) in *Neurospora*, which is generally cultured at 25°C. The pronounced stability of luciferase at 25°C limits its use as a reporter for dynamic transcription processes. Addition of a PEST sequence accelerates the turnover of luciferase in *Neurospora* by more than one order of magnitude. We show that the destabilized LUC-PEST protein (t_1/2_ ∼ 25 min) is a faithful reporter of promoter activity that readily uncovers rapid and considerably complex transcriptional dynamics.

## Materials and Methods

### Neurospora Strains and Culture Conditions

*Neurospora* strains carried the *ras-1^bd^* mutation [24]. For transformations, *ras-1^bd^; his-3* [25] and *ras-1^bd^; Δvvd; his-3* [15] were used. Standard growth medium contained 2% glucose, 0.5% L-arginine, 1× Vogel’s. When indicated, 10 µg/mL CHX was used.

### Plasmid Construction and Neurospora Transformation

*A Neuropsora* codon optimized PEST sequence (synthetized by GenScript) was inserted in front of the luciferase STOP codon in pFH62 *luc* (pBM60-*luc-trpC*, [26]) via a genetically engineered BglII site. The *frq* promoter and the *vvd* promoter, respectively, were amplified by PCR and inserted into via BamH1/NotI and EcoRI/NotI, respectively, into pFH62 lucPEST. *Neurospora* conidia were transformed as described [27]. The *his-3* locus was used for genomic integration of the plasmids by homologous recombination.

Codon optimized PEST sequence:

5′-TCC CAC GGC TTC CCC CCC GAG GTC GAG GAG CAG GCC GCC GGC ACC CTC CCC ATG AGC TGC GCC CAG GAG AGC GGC ATG GAC AGG CAC CCC GCC GCC TGC GCC AGC GCC AGG ATC AAC GTC TAA -3′

Primers:

frqprom_fwd 5′-aaaggatccgggatagcagagaacctcaatctc-3′,

frqprom_rev 5′-aaagcggccgcatcgacaatcgaattccgga-3′,

vvdprom_fwd 5′-tttgaattcggtgccattggtcttgggttg-3′,

vvdprom_rev 5′-gcggccgcggtgctggttatgagacagtg-3′.

### RNA Analysis

RNA was prepared with peqGOLD TriFAST (peqLab, Erlangen, Germany) and reverse transcribed with the Maxima First Strand cDNA Synthesis Kit (Fermentas). Transcript levels were analyzed by quantitative real-time PCR in 96-well plates with the StepOnePlus Real-Time PCR System (Applied Biosystems). TaqMan Gene Expression Master Mix (Applied Biosystems) and was used with gene-specific primers and probes [13].

### *In vivo* Luciferase Measurements

Sorbose medium containing 1× FGS (0.05% fructose, 0.05% glucose, 2% sorbose), 1× Vogels, 1% agarose, 10 ng/ml biotin and 150 µM firefly luciferin was used for the assessment of the luciferase rhythms. 96-well plates were inoculated with 3×10^4^ conidia per well and incubated in DD at 25°C. Bioluminescence was recorded in DD or LD at 25°C with an EnVision Xcite Multilabel Reader (Perkin Elmer). The light intensity used in LD cycles was 40 µE.

**Figure 1:**
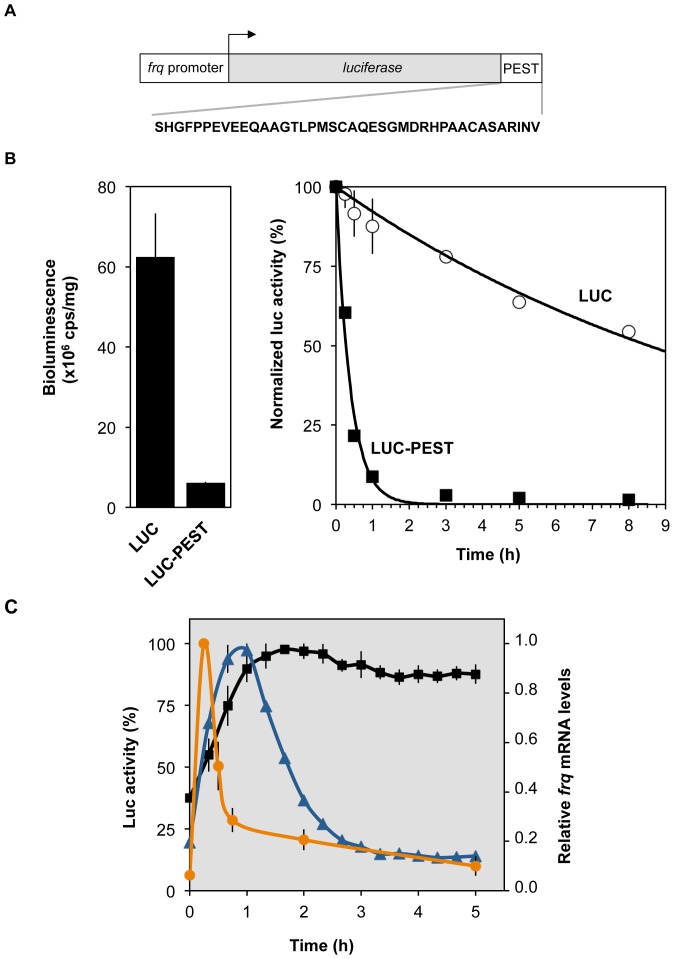
A PEST signal reduces luciferase stability. (**A**) Schematic representation of the luciferase reporter expressed under control of the promoter of the *frequency* gene (*frq promoter*). The coding region of the firefly luciferase has been codon optimized for *Neurospora crassa* expression [19]. The PEST sequence fused to the 3′-end of the luciferase is indicated. The reporter gene was integrated into the genome downstream of the his-3 locus. (**B**) A protein stability assay in light grown cells was carried out in the presence of cycloheximide (CHX) to compare the degradation of LUC-PEST and unmodified LUC. Mycelia were grown in liquid culture at 25°C and CHX was added at t  = 0 at a final concentration of 10 µg/ml. Cells were harvested at the indicated time points, native protein extracts were prepared and luciferase activity was analyzed as described in Material and Methods. (Left) Absolute bioluminescence levels at t  = 0 are shown. (Right) Bioluminescence levels are blotted over time. Levels at t  = 0 were set to 100% (Error bars indicate to ± SEM, *n*  = 3). Exponential trend lines are fitted to the data (values <5% were excluded). (**C**) Cultures of *frq::luc* (blue line) and *frq::luc-PEST* (orange line), grown in darkness under growth-restricting conditions on solid medium in a 96-well plate, were exposed to a 1 min light pulse and then released into constant darkness. Luciferase activity was recorded *in vivo* for 5 hours in 20 min intervals. *frq* mRNA levels (black line) were determined by qPCR in *WT* strain grown in liquid medium. Data were normalized by setting the maxima to 100%. (Error bars indicate ± SEM, *n*  = 3).

Light pulse assay (Fig. 1C): liquid standard growth medium contained 2% glucose, 0.5% L-arginine, 1× Vogel’s and 150 µM firefly luciferin. 96-well plates were inoculated with 1.5×10^5^ conidia per well and incubated in DD for 1 day at 25°C. Plates were then exposed to a 1 min light pulse (80 µE) before bioluminescence was recorded in DD at 25°C.

### *In vitro* Luciferase Measurements

10 µl protein extract (10 µg/µl), prepared as described [27], and 30 µl reaction buffer (10 mM luciferin, 3 mM ATP, 15 MgSO_4_, 50 mM Hepes pH 7.5) were combined in a 96-well plate and bioluminescence was measured with an EnVision Xcite Multilabel Reader.

## Results

### A PEST Signal Destabilizes Luciferase in Neurospora

In order to decrease the half-life of firefly luciferase (LUC) a PEST-encoding DNA template [23] was codon optimized for *Neurospora* and fused to the 3′ end of the codon-optimized *luc* ORF [19]. The *luc-PEST* fusion gene was expressed in *Neurospora* under the control of the *frequency* (*frq*) promoter (Fig. 1A). LUC-PEST was expressed at ∼10-fold lower level than unmodified LUC (Fig. 1B left), suggesting that the protein is unstable. To assess the destabilizing effect of the PEST-tag liquid cultures of strains expressing LUC and LUC-PEST, respectively, were treated with the translation inhibitor cycloheximide (CHX). Mycelia were then harvested over a time course of 8 h and luciferase activity (bioluminescence) was measured *in vitro* in protein extracts prepared from these samples. Under these conditions LUC-PEST was rapidly degraded with a half-time (t_1/2_) of ∼ 25 min while unmodified LUC was slowly degraded with an apparent t_1/2_>8 h, (Fig. 1B right). Thus, the PEST-tag accelerates the turnover kinetics of the protein by more than one order of magnitude.

The rapid degradation of LUC-PEST prompted us to investigate the response of the *frq* promoter to a short light pulse (80 µE, 1 min). *frq* mRNA peaked about 15 minutes after the light pulse and decreased to baseline levels after ∼ 2 h (Fig. 1C, orange line). Bioluminescence supported by *frq::luc-PEST* reached its peak 40–60 minutes after the light pulse and then decreased to baseline levels after ∼ 3.5 h. In contrast, the activity of the stable luciferase supported by *frq::luc* peaked after 2 h and levels did not drop significantly over the course of five hours. The data highlights the usefulness of both, stable and unstable reporters. The *frq*::*luc-PEST* reporter activity reflects with a rather short delay the transcriptional dynamics of the *frq* promoter, while the activity of *frq*::*luc* corresponds, due to the pronounced stability of LUC, to a temporal integration of the produced mRNA molecules.

### Free-running frq Transcription Rhythms

**Figure 2:**
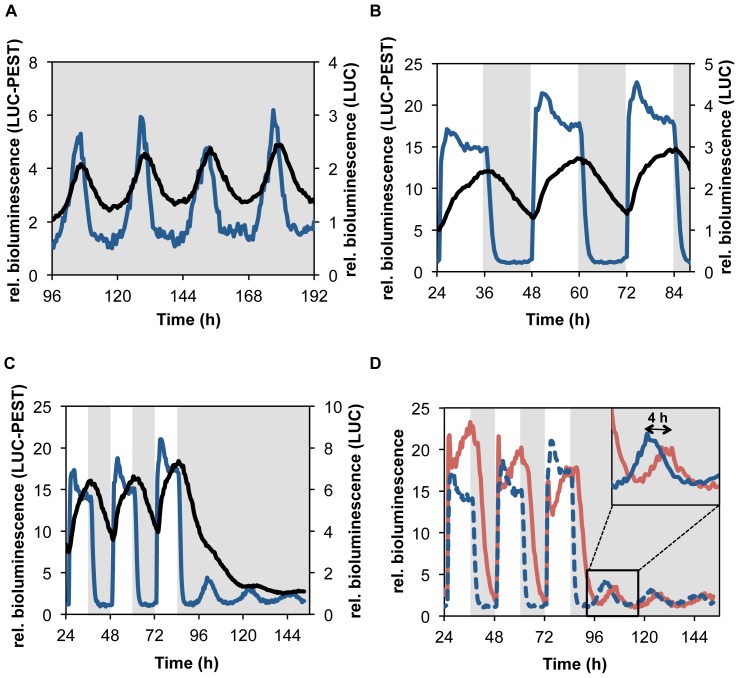
*frq*-promoter driven luc-PEST exhibits high amplitude oscillations. *frq::luc* (black lines) and *frq::luc-PEST* (blue lines) strains grown in 96-well plates were synchronized with a 1 hour light pulse and luciferase activity was measured every 15 minutes under the indicated conditions: (**A**) constant darkness (DD), (**B**) 12 h:12 h light/dark cycles (LD12∶12) and (**C**) 4 days in LD12∶12 followed by 3 days in DD. Representative bioluminescence records (starting at day 2) of individual cultures are shown. Data were normalized by setting the minima to 1. (**D**) The *frq::luc-PEST* reporter conveniently resolves the known differences in transcription dynamics between *WT* (blue) and Δ*vvd* (red) strains. The *frq::luc-PEST* reporter was expressed in a Δ*vvd* strain, which was exposed to the same light-dark regime as above. The *frq::luc-PEST* activity of *WT* grown in the same plate from part (**C**) is shown again for comparison (dotted line). Insert: magnification showing the ∼4 h phase delay of Δ*vvd* after release to DD [34].

We compared the circadian luciferase activity rhythms supported by the *frq*::*luc* and *frq*::*luc-PEST* reporters. The corresponding strains were cultured under restricted growth conditions (sorbose-containing solid growth medium), which allow life-cell measurements over extended time periods in a 96-well plate format. In constant darkness (DD) both reporters oscillated robustly with a free running period of ∼ 23 h (Fig. 2A). However, the amplitude (peak:trough ratio) supported by *frq*::*luc-PEST* was substantially higher than the amplitude of the *frq*::*luc* strain (6-fold versus 2.3-fold). Thus, the *luc-PEST* reporter appears to be more sensitive and may thus be suitable for recording of low amplitude promoter activity rhythms that would be masked by stable luciferase.

### Entrained frq Transcription Rhythms

A key property of circadian clocks is their responsiveness to environmental cues such as light [28], [29], [30]. We recorded the activity of *frq*::*luc-PEST* and *frq*::*luc* strains grown under a 12 h light/12 h dark (LD) cycle (Fig. 2B). In this artificial square wave light/dark regime LUC-PEST activity reached a maximum rapidly after lights-on and then adapted within a few hours at a high steady-state levels. After light-to-dark transition LUC-PEST levels decreased quickly, reaching trough levels about 2–3 h after lights-off. The peak:trough expression ratio supported by *frq::luc-PEST* was ∼ 20-fold. This activity profile of *frq*::*luc-PEST* corresponds closely to the previously reported temporal expression profile of *frq* RNA in light/dark cycles [31], [32], indicating that the destabilized luciferase is a faithful reporter of the transcription dynamics of the *frq* promoter. In contrast, the activity of the stable luciferase encoded by *frq*::*luc* increased steadily throughout the light phase and then decreased during the dark phase. The peak:through ratio of the luc activity was about 2-fold. The temporal activity profile of *frq*::*luc* does not resemble the transcription dynamics the *frq* promoter.

### Transition from Entrained to Constant Conditions

Transcript levels supported by the *frq* promoters are substantially higher in light than in the dark [33]. We recorded with *frq::luc-PEST* and *frq::luc* reporter strains the transition from light/dark entrained conditions to constant darkness. Luciferase activity of the strains was measured for 4 days in 12 h/12 h LD cycles followed by 3 days in DD. The *frq::luc-PEST* reporter impressively revealed differences in the expression levels and amplitude of *frq* promoter driven expression under entrained and free running conditions (Fig. 2C). Thus, the *luc-PEST* reporter allows analyzing temporal expression profile of the *frq* promoter over an extended time period under varying conditions. In contrast, the rapid shutdown of *frq* transcription after transition from entrained to constant conditions was not resolved by the *frq::luc* strain (Fig. 2C). Due to the slow degradation of the stable LUC that had accumulated at high levels during the preceding light phase the free-running *frq* promoter oscillations in DD was almost completely masked.

### Frq promoter Dynamics in *WT* versus Δ*vivid*

VVD determines the phase of the circadian clock by inhibiting the light-activated WCC in particular after light-to-dark transition of *Neurospora* [34]. We analyzed the dynamics of *frq::luc-PEST* expression in a Δ*vivid (*Δ*vvd*) strain under the same LD and DD regime as for *WT*. Following light-to-dark transitions, LUC-PEST activity decreased more slowly in Δ*vvd* than in *WT*, which perpetuated in constant conditions into a 4 h phase delay (Fig. 2D). Hence, considerably small differences in circadian promoter activity are readily and reliably detected by the *luc-PEST* reporter. The high temporal resolution of the *frq::luc-PEST* reporter revealed in addition complex differences between Δ*vvd* and *WT* in the adaptation dynamics of the *frq* promoter during the light phase in (Fig. 2D), which will be further investigated elsewhere.

### Transcription Dynamics in Complex Light Regimes

The blue-light photoreceptor VVD regulates sensory adaptation of *Neurospora* to light by inhibiting the light-activated WCC [17], [32], [35], [36], allowing *Neurospora* to respond to changes in light intensity over several orders of magnitude.

**Figure 3:**
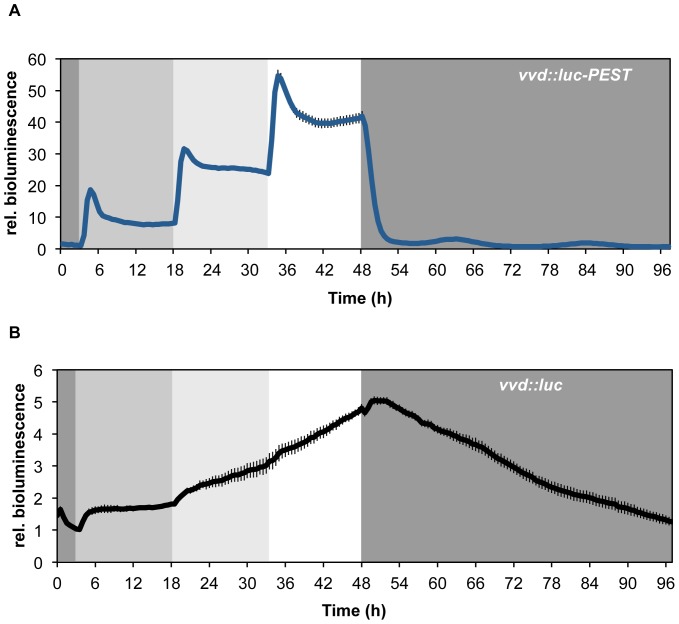
Light response and adaptation of *vvd*-promoter driven luciferase expression. *Luc* and *luc-PEST* were expressed under the control of the *vvd* promoter. Cultures were subjected to three consecutive 15 h light steps with increasing intensity (0.35 µE, 3.5 µE and 30 µE) followed by 48 h of constant darkness. Luciferase activity was measured in 30 min intervals. (**A**) *vvd::luc-PEST* (n = 20) (**B**) *vvd::luc* (n = 4). Error bars indicate ± SEM.

To analyze the temporal dynamics of the *vvd* promoter, we expressed *vvd::luc-PEST* and *vvd*::*luc* reporters in a *WT* background. Dark-grown conidial cultures of *vvd::luc-PEST* and *vvd*::*luc* were exposed in 96-well plates to consecutive 15 h light-intensity steps of 0.35 µE, 3.5 µE and 30 µE followed by a final 48 h period in the dark. As shown for *vvd::luc-PEST*, light responses of 20 individual cultures were highly reproducible (Fig. S1A, B). Consistent with *vvd* mRNA data obtained from a similar experimental setup [17], LUC-PEST activity increased sharply after each light step followed by an adaptation phase and approached a rather constant steady-state level that correlated with light intensity (Fig. 3A). Expression levels dropped rapidly after the subsequent light-to-dark transition and then oscillated at a low level in circadian fashion. The data demonstrates that *Neurospora* quickly responds to increases as well as decreases in light intensity, adjusting expression levels of *vvd* according to ambient light. In contrast, due to the stability of the luciferase, bioluminescence traces of a *vvd*::*luc* strain failed to resolve the induction and adaptation responses of the *vvd* promoter to the light intensity steps and to the final dark-transition (Fig. 3B).

## Discussion

Destabilized luciferase reporters have been successfully used in a number of circadian as well as non-circadian studies [37], [38], [39], [40], [41]. In *Neurospora*, a codon-optimized luciferase has been used as transcriptional reporter of circadian promoter activity and as translational fusion with the FRQ protein to monitor clock protein rhythms [42]. We have shown here that in *Neurospora* firefly luciferase (LUC) activity is stable (t_/1/2_ ∼8 h). The same protein in mammalian cells is turned over significantly faster (t_/1/2_ ∼4 h). Since LUC is a thermo-labile protein, it is likely that the differences in LUC stability are due to the growth temperatures, i.e 25°C for *Neurospora* and 37°C for mammalian cells. The considerable stability of LUC at 25°C limits its use as transcription reporter, particularly as a reporter of rapid transcription dynamics, in *Neurospora* but presumably also in other organisms that grow at lower temperatures. A PEST signal fused to LUC tremendously destabilizes the protein, triggering its degradation in *Neurospora* with a half-time of only ∼25 min. The unstable luciferase is a faithful reporter of promoter activity, resolving rapid as well as small changes in gene transcription, which would be masked by a stable LUC reporter. The LUC-PEST reporter system in combination with growth restricted culture conditions makes it possible to record promoter activity *in vivo* over several days. In combination with a plate-reader equipped with an automated stacker, the system is suitable for medium to high throughput promoter analyses in 96-well plate formats. The high-density time course analyses are highly reproducible even under complex physiological regimes (e.g. light-dark cycles or light steps). The system is thus superior to conventional approaches based on labor intensive and rather error-prone quantification of RNA expression by qPCR or Northern analysis.

## Supporting Information

Figure S1 **Light response and adaptation of** ***vvd*** **-promoter driven luciferase expression is highly reproducible.** Data from the experiment performed in Fig. 3A. 20 separate traces are shown as **(A)** raw data measured in bioluminescence counts per second **(B)** normalized data. Traces were normalized to the mean bioluminescence levels of each trace.
(PDF)
