# Supplementary figures and images for: Light-Dependent and Circadian Transcription Dynamics In Vivo Recorded with a Destabilized Luciferase Reporter in Neurospora
Source: PLoS One. 2013 Dec 31;8(12):e83660. doi: 10.1371/journal.pone.0083660 (PMC3877077; doi:10.1371/journal.pone.0083660)

**A**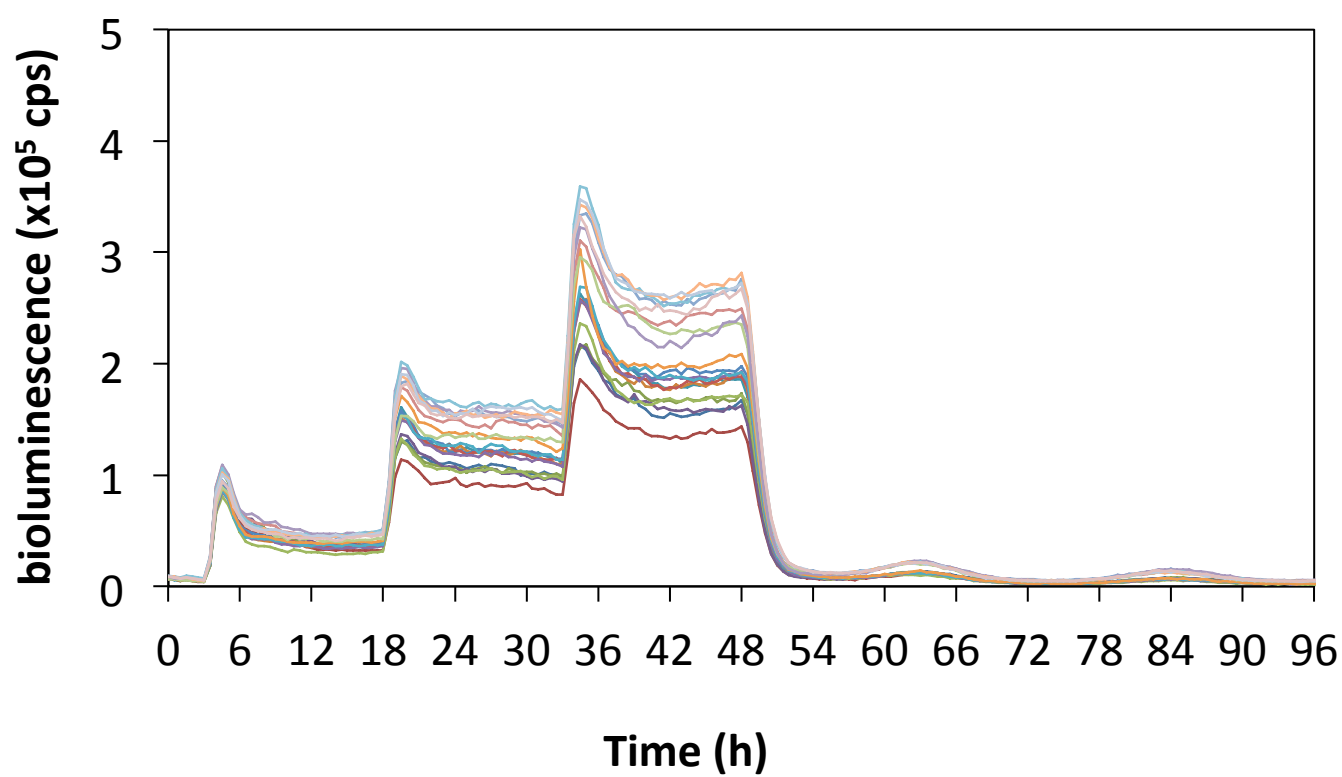**B**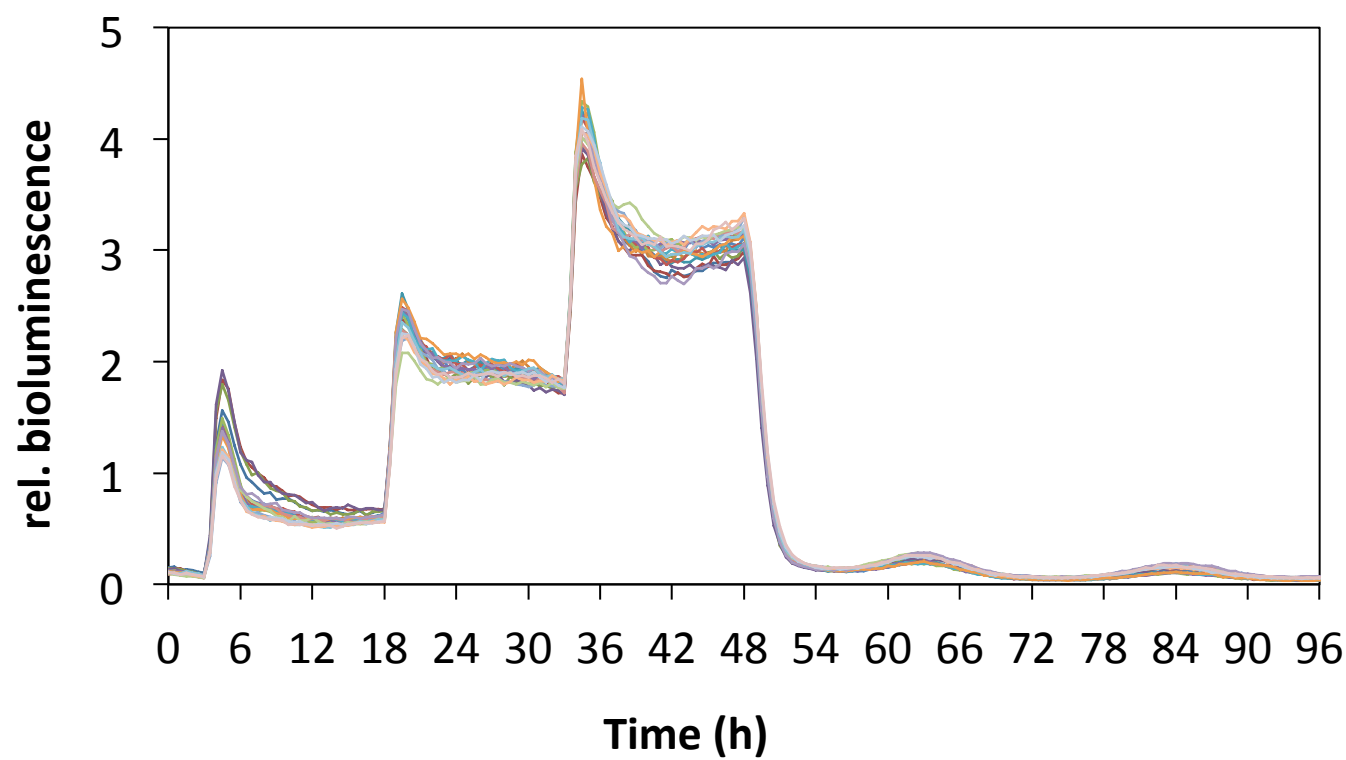

Supplement: Figure S1 — Light response and adaptation of vvd -promoter driven luciferase expression is highly reproducible. Data from the experiment performed in Fig. 3A. 20 separate traces are shown as (A) raw data measured in bioluminescence counts per second (B) normalized data. Traces were normalized to the mean bioluminescence levels of each trace. (PDF) [file pone.0083660.s001.pdf]
